# Three-Dimensional Weld Pool Monitoring and Penetration State Recognition for Variable-Gap Keyhole Tungsten Inert Gas Welding Based on Stereo Vision

**DOI:** 10.3390/s24237591

**Published:** 2024-11-27

**Authors:** Zishun Wang, Yonghua Shi, Yanxin Cui, Wenqian Yan

**Affiliations:** 1 School of Mechanical and Automotive Engineering, South China University of Technology, Guangzhou 510640, China; 202010100194@mail.scut.edu.cn (Z.W.); 202220100609@mail.scut.edu.cn (W.Y.); 2 Guangzhou Railway Polytechnic, Guangzhou 511300, China; cuiyanxin@gtxy.edu.cn

**Keywords:** K-TIG welding, three-dimensional weld pool monitoring, penetration recognition, stereo vision

## Abstract

K-TIG welding offers the advantages of single-sided welding and double-sided formation, making it widely used for medium/thick-plate welding. The welding quality of K-TIG is closely linked to its penetration state. However, the assembly gap in medium/thick-plate workpieces can easily result in an unstable penetration state. In K-TIG welding, the geometric characteristics of the weld pool are closely related to the penetration state. Compared to arc voltage sensing and acoustic signal sensing, visual sensing is a method capable of obtaining the three-dimensional geometric features of the weld pool. To this end, a K-TIG weld pool three-dimensional monitoring algorithm based on a semantic segmentation network using a stereo vision system with a single High-Dynamic-Range (HDR) camera is proposed in this paper. In order to identify the assembly gap of medium/thick-plate workpieces, a gap width extraction algorithm based on the watershed method is proposed. Subsequently, a penetration state recognition model is constructed, taking the three-dimensional geometric features of the weld pool and the gap width as inputs, with the penetration state as the output. The relationship between the input features and the accuracy of penetration recognition is analyzed through feature ablation experiments. The findings reveal that gap width is the most critical feature influencing the accuracy of penetration recognition, while the area feature negatively affects this accuracy. After removing the area feature, the accuracy of the proposed penetration recognition model reaches 96.7%.

## 1. Introduction

Keyhole Tungsten Inert Gas (K-TIG) welding achieves deep penetration by increasing the input current, building on the principles of traditional TIG welding. It offers the advantages of single-sided welding with double-sided formation, without the need for beveling (plate thickness ≤14 mm), and is widely used in the connection of medium/thick metal plates [1], such as in oil storage tanks. To enhance production efficiency and address the welder shortage, the automation of K-TIG welding has become a key focus of development [2]. And the ability to recognize penetration is one of the most important features that an automated K-TIG welding system should have [3]. The penetration states in K-TIG welding can be categorized into three types: under-penetration, good penetration, and over-penetration. Among these, both under-penetration and over-penetration result in a K-TIG welding quality that can not meet quality standards. When welding medium/thick plates with large dimensions, workers use lifting equipment and traction ropes to assemble the workpieces. The instability of manual operations can easily result in variable assembly gaps, which is the primary factor causing unstable penetration states in K-TIG welding. Therefore, this paper proposes a penetration state recognition model designed for variable-gap K-TIG welding.

In K-TIG welding, the features of the weld pool are closely related to the penetration state [4]. And the features of the weld pool can be monitored by arc sensing, acoustic sensing, and vision sensing. Arc sensing has the advantages of simplicity. Zou et al. used an arc sensor to monitor the weld pool surface in Pulse Gas Metal Arc Welding (GMAW-P) and developed an estimation model for penetration depth [5]. Acoustic sensing can capture rich information from the inside of the weld pool. Cui et al. combined arc sensing and acoustic sensing to recognize the penetration state in K-TIG welding, developing an ECOC-SVM-GSCV model that achieved an accuracy of 98.7% [3]. Vision sensing offers the advantages of intuitiveness and robustness, attracting the attention of researchers.

Based on the use of a light source, vision sensing can be categorized into active vision and passive vision. In the context of weld pool monitoring, the light source for active vision is typically a laser. Cheng et al. projected a laser stripe onto the weld pool in Gas Tungsten Arc Welding (GTAW) and developed a Convolutional Neural Network (CNN)-based penetration prediction model that achieved an accuracy of 97.5% [6]. Huang et al. employed a grid-structured laser to reconstruct the three-dimensional topography of TIG welding, observing the changes in the weld pool as droplets entered it [7]. Jeon et al. combined an infrared camera and a laser line scanner to develop a weld pool depth estimation model that achieved an accuracy of 25.97 µm [8]. While active vision-based weld pool monitoring offers advantages in precision and robustness, the complexity of the laser system can make it challenging to implement in welding manufacturing. In contrast, passive vision is more cost-effective and simpler to use.

In passive vision-based weld pool monitoring, the extracted features of the weld pool include both planar and three-dimensional (3D) geometric features. The planar geometric features primarily consist of area, width, and length. Liu et al. proposed to used a morphological image processing algorithm to monitor the width and length of the weld pool in laser welding [9]. Huang et al. also proposed a traditional image processing algorithm to monitor the weld pool geometry in Gas Metal Arc Welding (GMAW), incorporating the advancing contact angle as an additional planar feature [10]. Andres Giron-Cruz et al. employed the shadowgraphy technique to monitor the width, height, and oscillation frequency of the weld pool in GMAW. They developed a controller that fuses fuzzy logic with an artificial neural network to regulate penetration [11]. Xiong et al. utilized Canny edge detection to extract the width of the weld pool in Gas Tungsten Arc Additive Manufacturing (GTA AM) and developed a parameter self-adjusting fuzzy controller to regulate the layer width, achieving an accuracy of 0.5 mm [12]. Similarly, Zhang et al. proposed a pixel-based algorithm to extract the length of the weld pool in AM [13]. While traditional image processing algorithms offer advantages in simplicity and low computational requirements, their stability can be easily affected by factors such as arc light. In contrast, deep learning-based algorithms are more resilient to interference in welding environments. Asadi et al. proposed using the You Only Look Once (YOLOv8) network to segment the weld pool area and developed a CNN model that takes the segmentation mask as input and outputs the width, height, and area of the bead [14]. Cai et al. proposed using a lightweight semantic segmentation network, DeepLab-m, to segment the weld pool area in laser welding, and they concatenated the segmentation results to monitor the formation [15]. Baek et al. employed a semantic segmentation network to obtain the masks of the GTAW weld pool and used these segmentation masks as input to a CNN to estimate the penetration depth, achieving a mean absolute error of 0.0516 mm [16]. To improve the accuracy of estimating the geometric features of the bead, Jamnikar et al. utilized a multi-modality CNN that takes weld pool images and thermal profiles as inputs to estimate the geometric features of the bead in wire-feed laser additive manufacturing (WLAM) [17].

Researchers often use planar geometric features to estimate the 3D characteristics of the weld pool or its penetration state, recognizing that 3D features are closely related to penetration. Consequently, some researchers attempted to monitor 3D features directly. However, the weld pool behaves like a mirror, posing significant challenges for passive vision-based 3D reconstruction. Chen et al. utilized the mirror similarity of the weld pool and proposed to use the reversed electrode image to monitor the 3D geometry of the weld pool [18]. The proposed algorithm relies on the reflection of the electrode and therefore can be easily affected by the angle of the camera. Neill et al. used a pair of synthetic images generating from a weld pool simulation model to validate the efficiency of stereo vision 3D reconstruction on the weld pool, but they did not consider the mirror property [19]. Liang et al. designed a biprism stereo vision system with a single camera and used it to reconstruct the 3D weld pool surface in GMAW-P with a corner detection algorithm [20]. Similarly, Xiong et al. used the biprism stereo vision system to monitor the 3D weld pool in Gas Metal Arc Additive Manufacturing (GMA AM) and built up a fuzzy controller to adjust the current, achieving a consistent layer width with an accuracy of 3% [21]. However, the stereo matching algorithm they used is traditional, and it is difficult for its accuracy and stability to meet the requirements of welding. Therefore, Zhang et al. used the same stereo vision system as Liang’s to build a 3D weld pool monitor system with a deep learning-based neural network ACVNet and built up a penetration state classifier MobileNetV3 with an accuracy of 99.83% [22].

Zhang has shown the power of a deep learning algorithm in stereo vision-based 3D weld pool monitoring [22]. However, in welding applications, it is hard to train a stereo matching network (ACVNet for example) with a precise disparity label. Therefore, in this paper, in order to realize penetration state recognition for variable-gap K-TIG welding, a 3D weld pool surface monitoring algorithm is developed based on a stereo vision system with a single camera proposed previously [23]. Firstly, given that the calculation of the weld pool’s 3D geometric features relies solely on its contours and the challenges in obtaining precise disparity labels for a 3D weld pool, a semantic segmentation network is employed to segment the weld pool area and reconstruct its 3D contours. Secondly, to extract the gap width, which is also closely related to the K-TIG penetration state, a watershed-based algorithm is proposed. Finally, a penetration state classifier is developed, and the significance of the input features for the accuracy of the penetration recognition model is analyzed.

## 2. Experimental System and Dataset

**Figure 1:**
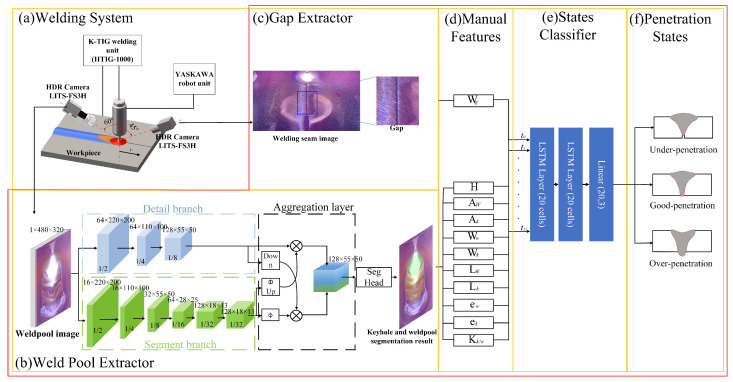
Schematic of K-TIG welding penetration recognition model.

The schematic of the proposed K-TIG welding penetration recognition model is shown in Figure 1. The welding system consists of a K-TIG welding unit, a YASKAWA robot unit, an industrial computer, and two HDR cameras. The torch is carried by the YASKAWA robot, with the HDR cameras fixed to the torch. One camera is placed at the front of the welding torch with an angle of 45 and a distance of 260 mm. The other is fixed behind the welding torch, creating a stereo vision system with a single camera to monitor the three-dimensional geometric features of the weld pool. And the angle is 60 with a distance of 230 mm. The parameters of the camera are listed in Table 1. The lens is MVL-MF2528M-8MP with a focus length of 25 mm. And the HDR cameras, the Tool Center Point (TCP) of the robot, and the Eye-in-Hand matrix are calibrated.

**Figure 2:**
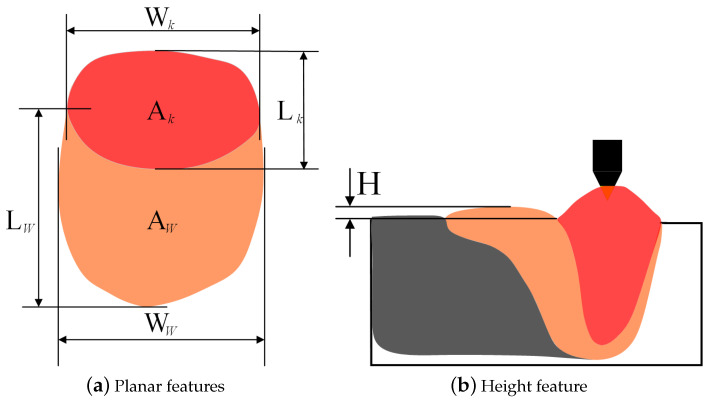
Definition of weld pool features.

In the weld pool extractor, a semantic segmentation network BiSeNetV2 [24] is selected to segment the weld pool area in the weld pool images so that the geometric features can be extracted. The geometric features include planar features including area (A), width (W), length (L), eccentricity (e=L/W), and the keyhole-to-weld-pool ratio ($K_{k/w}=A_{k}/A_{w}$), as shown in Figure 2a, and the height feature (H), as shown in Figure 2b. In the gap extractor, a gap width extraction algorithm based on watershed is proposed. And the aforementioned features will be input into the K-TIG penetration state classifier to recognize the penetration state. A long short-term memory (LSTM) network [25] with two hidden layers is selected as the penetration state classifier.

**Figure 3:**
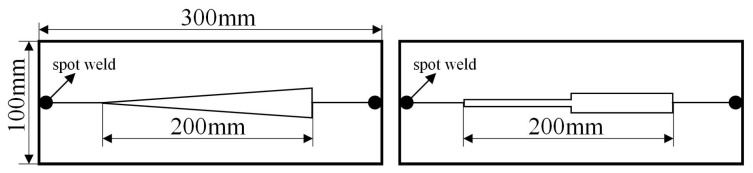
Two different types of gaps.

In order to facilitate the design of a variable-gap K-TIG penetration state recognition model, a corresponding dataset is constructed. In the application scenario of medium/thick plates, the assembly gap is the main factor affecting the penetration state of K-TIG welding. The assembly gap can be mainly divided into two types, namely gradual gap and mutant gap. The gradual gap is mainly caused by the inconsistency of the front and rear fit of the workpiece when the workers use traction ropes to assemble the workpieces. And the mutant gap mainly occurs at the connection between two workpieces. There might be an assembly deviation between the two adjacent workpieces causing a mutant gap. In this paper, the material of the workpieces is Q345R with a thickness of 10 mm, which is a commonly used material in the manufacturing of oil storage tanks. The parameters of the gaps are shown in Figure 3 and Table 2. The welding current is 500 A and the welding speed is 4 mm/s. The protection gas is pure Argon with a flow rate of 20 L/min.

In stereo vision, due to the different reflections of arc light on the weld pool surface under different viewing angles, it is difficult to align the keyhole entrance under the two viewing angles. Therefore, in this paper, the planar features of the weld pool are extracted only based on the left view. The height features are extracted by stereo vision. For the planar features, one image is taken for annotation at an interval of 10 frames in each group of experiments. Together with the previous dataset, there are a total of 963 annotated images. In total, 80% of all images are randomly selected to form a training set (736 images), and the remaining form a validation set (227 images). For the weld pool height feature, images from the left and right view are taken for annotation at an interval of 20 frames in each group of experiments, and only the contour of the weld pool will be annotated. There are a total of 682 annotated images in the training set and 162 images in the validation set. For the K-TIG penetration state classifier, all experiments contain a total of 3693 data points, of which 1290 are under-penetration, 1990 are good penetration, and 413 are over-penetration. In total, 20% of all the data are randomly selected as the test set, 20% are used as the validation set, and the remaining are used as the training set.

## 3. Feature Extraction

### 3.1. Three-Dimensional Geometric Features of the Weld Pool

In the extraction of the three-dimensional geometric features of the weld pool, both planar features and the height feature are extracted after segmentation by BiSeNetV2. The BiSeNetV2 is trained by the Stochastic Gradient Descent (SGD) algorithm with a momentum of 0.9. The batch size is set as 32. The weight decay is 0.0001. The initial learning rate is 0.01, with a “poly” learning rate strategy. The loss function is Focal Dice Loss [4], proposed previously. The epochs are set to 300. The training results are shown in Table 3.

#### 3.1.1. Planar Feature Extraction

**Figure 4:**
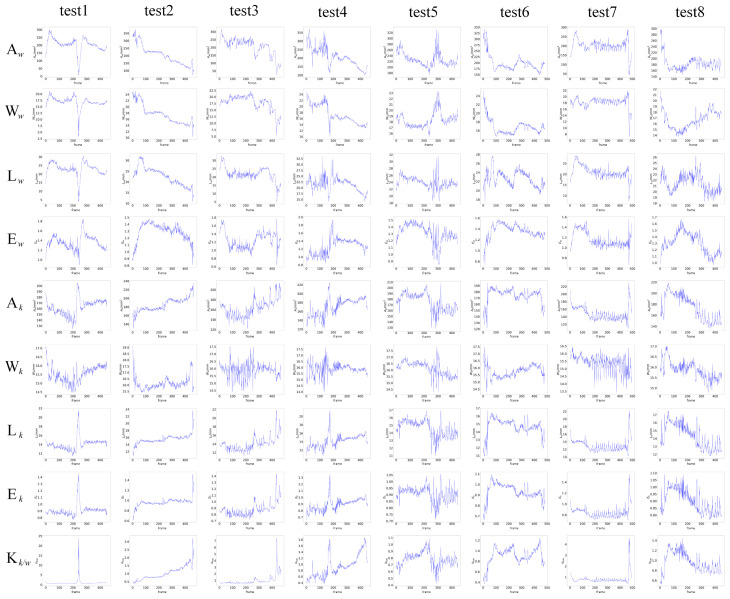
The extracted planar features of the weld pool.

After the weld pool image is segmented by BiSeNetV2, the keyhole area and the weld pool area can be obtained. The keyhole feature is processed in the same way as the weld pool feature. Taking the keyhole area as an example, firstly, the maximum connected domain of the keyhole mask is retained to avoid the possible outlier misidentification points affecting feature extraction. Secondly, the mask image is rotated to be parallel to the *Y* axis of the image coordinate according to the calibration result, to facilitate the extraction of the length and width of the keyhole by the line-scanning algorithm. The area is calculated once for each line. The area feature of the keyhole is the result of the accumulation of all lines. The extraction results are shown in Figure 4.

#### 3.1.2. Height of Weld Pool

**Figure 5:**
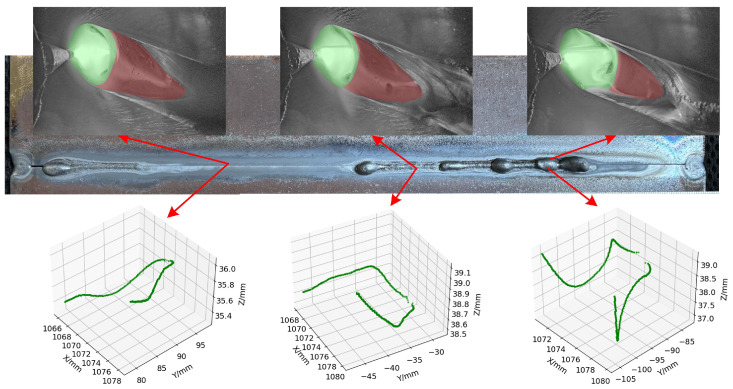
Limitations of planar weld pool features.

Planar features can only provide two-dimensional information of the weld pool, while the height information is also closely related to the penetration state. For example, the weld pool image with gaps in the good-penetration state is similar to the image without gaps in the under-penetration state, resulting in a similarity of planar features, while the height information can distinguish between the under-penetration state (0.7 mm) and the good-penetration state (0.5 mm), as shown in Figure 5. Without the support of height information, the penetration state recognition model may misclassify the under-penetration state as the good-penetration state.

**Figure 6:**
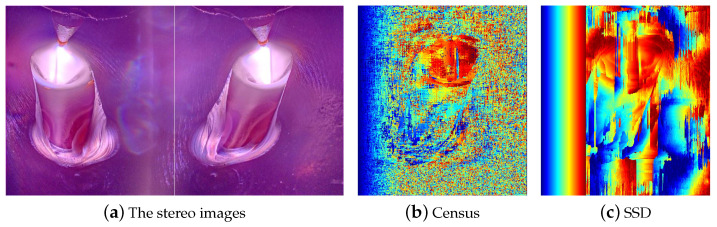
Weld pool image disparity results of traditional operators.

Therefore, in this paper, a stereo vision-based weld pool height extraction algorithm is proposed. The height feature depends on stereo vision-based three-dimensional weld pool reconstruction, and the most important step of stereo vision three-dimensional reconstruction is stereo matching. However, the surface of molten metal is similar to a mirror, and the pattern in the images is the reflection of the torch and tungsten needle. In addition, due to the different reflection angles in the left and right view, the textures in different views are totally different, which makes it difficult to perform stereo matching with traditional operators like Census [26] and SSD [27]. Figure 6b,c are the disparity maps obtained by the traditional stereo matching operators, in which the red color represents a large disparity. Due to the weak texture of the weld pool image, it is difficult for the traditional operators to perform stereo matching accurately.

**Figure 7:**
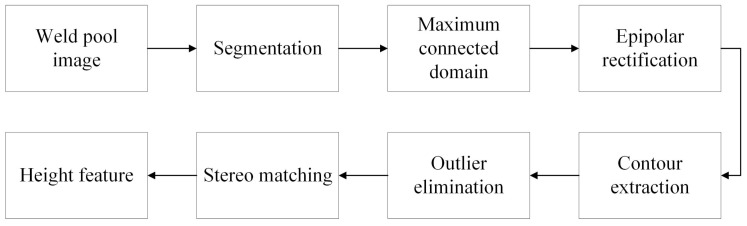
Flowchart of the weld pool height extraction algorithm based on stereo vision.

**Figure 8:**
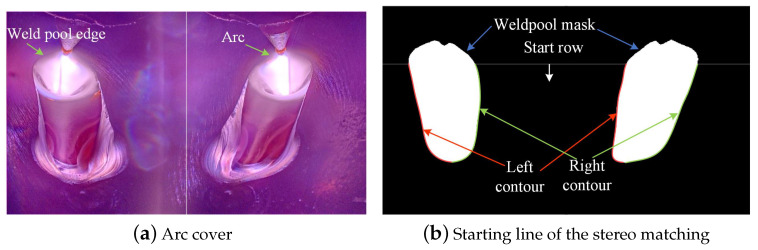
Setting the starting line of the stereo matching.

In order to avoid the difficulty of stereo matching caused by the mirror surface of the weld pool, in this paper, BiSeNetV2 is used to extract the edge of the weld pool and the three-dimensional reconstruction of the weld pool contour is realized. The specific steps of the algorithm are shown in Figure 7:

(1) The semantic segmentation network BiSeNetV2 is used to extract the contours of the weld pool under two views, and the largest connected domain of the segmentation mask is retained to prevent the mis-segmented areas from affecting subsequent recognition.

(2) Epipolar correction is performed on the segmentation mask, and the start row of stereo matching is set. Since the arc is a solid ion, it is blocked in the left and right views, resulting in the unreliable weld pool contour of the arc coverage area, which should not be used for stereo matching. The matching start row is set to be 300, as shown in Figure 8.

(3) The weld pool contour is extracted by scanning row by row. The operator [−1,1] is used to convolve with the segmentation mask to extract the left contours. And the operator [1,−1] is used to convolve with the segmentation mask to extract the right contours.

(4) The outliers of the contours should be eliminated. Some burrs may occur in the segmentation results and are mainly concentrated at the ends of the contour. Therefore, the extracted points are scanned from the middle of the contour to the two ends. If the distance between a point and its neighbors is greater than the set threshold (three pixels), the point will be eliminated and supplemented by linear interpolation.

(5) Stereo matching is performed. Since both the left and right views are contours, stereo matching is simple. Points of left or right contour under the same polar line are a pair of matching points.

(6) The three-dimensional coordinates of the weld pool contour in the camera coordinate system are calculated by the stereo Formula (1), where $\left(x_{lc},y_{lc},z_{lc}\right)$ is the camera coordinate in the left view, $u_{l}$ and $u_{r}$ are the *x* image coordinates in the left and right view, respectively, $f_{lx}$, $f_{ly}$, and *f* are the calibrated focus length, and *B* is the length of baseline between two views. Finally, the height distance between the highest and the lowest point is calculated as the height feature of the weld pool.

(1) $$\left\{\begin{array}{c} x_{lc}=z_{lc}u_{l}/f_{lx}\\ y_{lc}=z_{lc}v_{l}/f_{ly}\\ z_{lc}=\frac{f\;\cdot \;B}{u_{l}-u_{r}} \end{array}\right.$$

### 3.2. Gap Width of Welding Seam

**Figure 9:**
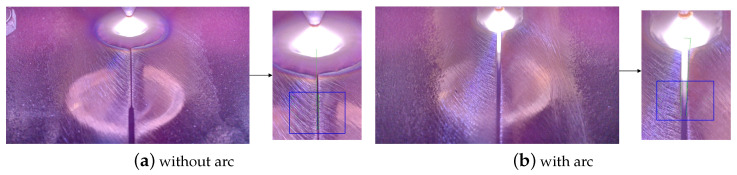
Gap width detection.

In order to extract the gap width of the welding seam, a gap width extraction algorithm based on the watershed algorithm is proposed. The welding seam is recognized by the algorithm proposed previously [28]. And the watershed segmentation algorithm is used to extract the gap by taking the position with the maximum or minimum pixel value of each row and both sides of the image as water injection points. As shown in Figure 9, the specific steps are as follows:

(1) A fixed RoI (Region of Interest) is set, as shown in the blue box in Figure 9. The welding seam is recognized by the algorithm proposed previously

(2) Bilateral filtering is performed on the RoI image to eliminate the influence of salt-and-pepper noise on the watershed segmentation algorithm.

(3) The maximum pixel value of each row of the recognized welding seam is counted. If the maximum pixel value is greater than 200 (there is arc light in the gap), the position with the maximum pixel value and the edges of the RoI image on both sides are used as water injection points. Otherwise, the position with the minimum pixel value and the edges of the RoI image on both sides are used as water injection points. After all the pixel are flooded, the last flooded point comprises the edges of the gap.

(4) Since the welding seam recognition algorithm uses global information, which is more reliable, the outliers of gap extraction should be eliminated according to the recognized welding seam. If the position with the maximum or minimum pixel value is on the left side of the welding seam, the right edge with a more than 5 pixel distance to the welding seam is eliminated. If the position with the maximum or minimum pixel value is on the right side, the left edge with a more than 5 pixel distance to welding seam is eliminated.

(5) Line fitting with RANdom SAmple Consensus algorithm (RANSAC) is performed on the left and right gap edge points, respectively. If difference of the slopes is larger than 1, the detection result of the previous frame is used. Otherwise, the intersection points with the upper and lower edges of the RoI are restored to the world coordinates through the homography transformation matrix (obtained by camera calibration), and the distance between them is calculated as the gap width. If the gap width is greater than the set threshold (2.5 mm), it will also be considered a detection failure and the detection result of the previous frame will be used.

(6) In order to further improve the stability of the algorithm, a queue with a length of 10 is used to perform median filtering.

## 4. K-TIG Penetration State Classifier

Based on the features extracted before, a K-TIG penetration state classifier with LSTM is constructed. Before training the classifier, it is necessary to determine the penetration state label and normalize and filter the feature data.

### 4.1. Penetration Label Determination

**Figure 10:**
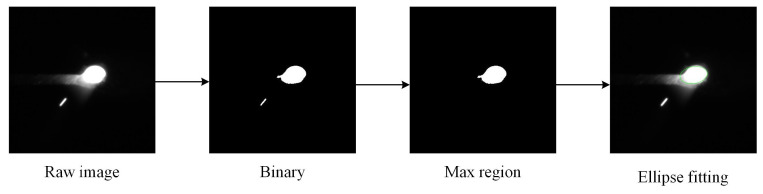
Width extraction of keyhole exit.

Training the penetration state recognition model requires a penetration state label. Thus, a Hikvision camera with an exposure time of 100 µs is set on the back of the workpiece to obtain the keyhole exit features corresponding to each frame of data. The width of the keyhole exit is extracted, as shown in Figure 10. The specific steps are as follows:

(1) The keyhole exit image is segmented with a threshold of 200. The largest connected domain is retained, and the contour of the segmentation mask is extracted.

(2) To avoid the interference of arc light, RANSAC ellipse fitting is performed on the contour.

(3) The world coordinates of the center point, major axis point *a*, and minor axis point *b* of the ellipse are calculated. According to the angle between the major axis and the *X* axis of world coordinate, the axis perpendicular to the welding direction (*Y*) is taken as the width. If the major axis is closer to the *X* axis, 2b is taken as the width; otherwise, 2a is taken as the width.

(4) The K-TIG penetration state determination rules are as follows: if the width of the keyhole exit is less than 1 mm or the back seam has no excess height, it is considered as under-penetration. If the width is larger than 1 mm but less than 2 mm and the back seam has an excess height of 1–2 mm, it is considered as good penetration. Otherwise, it is considered as over-penetration.

### 4.2. Data Normalization and Filtering

For deep learning, if the range gap between features is huge, gradient explosion or gradient vanishing problems may occur during network training. In the features extracted before, the range of area is much larger than for the other features, so the data need to be normalized to ensure that different features have similar ranges. The Z-score method is chosen to normalize the data, which is defined as follows:

(2) $$\hat{x}=\frac{x-\mu }{\sigma }$$

where μ and σ are the mean and standard deviation of the dataset, respectively, and *x* and $\hat{x}$ are the data before and after normalization.

In addition to the problem caused by the difference in the range of input features, the accuracy of the recognition model will be affected by the accuracy of feature extraction. To this end, a moving average filter (Weight Moving Average, WMA) is used to reduce the impact of feature extraction on the accuracy of the recognition model:

(3) $$x_{t}^{\prime }=\frac{\sum_{i}^{N}x_{t-i}}{N}$$

where $x_{t}^{\prime }$ is the filtered data, $x_{t-i}$ represents the data at time t−i before filtering, and *N* is the filter window length. In this paper, *N* is set to 10. Moreover, the data in the welding starting and ending stages do not belong to normal welding data. The data of 50 frames after the arc appears and 50 frames before the arc disappears should be removed during the training and inference of the penetration state recognition model.

### 4.3. The Training of K-TIG Penetration State Recognition Model

**Figure 11:**
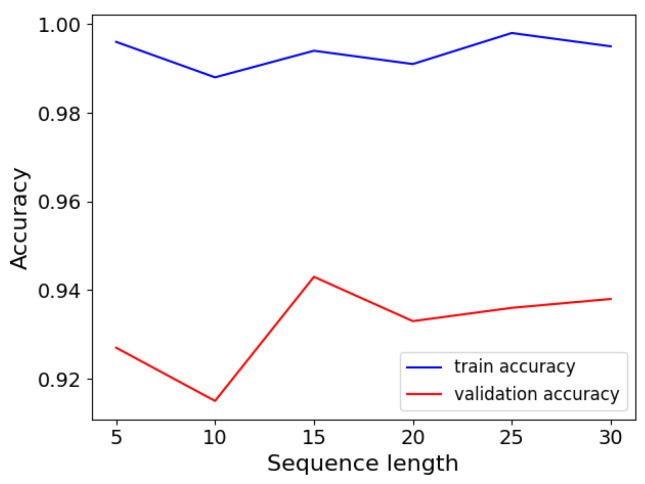
The training result of different sequence lengths in LSTM.

The LSTM model is trained with the three-dimensional features of the weld pool, the gap width, and the penetration label. The training setting of LSTM is similar to BiSeNetV2. The batch size is set to 64. The loss function is Mean Squared Error (MSE) and the epochs are set to 100. The length of the LSTM input sequence is a difficult hyperparameter to select because it is related to time. A short sequence cannot fully utilize the capabilities of LSTM, while a long sequence may lead to memory loss. Therefore, LSTM models with different sequence input lengths are trained as shown in Figure 11. According to the result, the input sequence length of LSTM is set to be 15.

## 5. Results and Analysis

In this paper, a variable-gap K-TIG penetration state recognition model based on the three-dimensional geometric features of the weld pool and the gap width is proposed. The three-dimensional geometric features of the weld pool can not only be used as the input features of the penetration state recognition model, but also be used to quantitatively characterize the quality of the weld formation.

### 5.1. Features of Weld Pool

#### 5.1.1. Planar Feature

**Figure 12:**
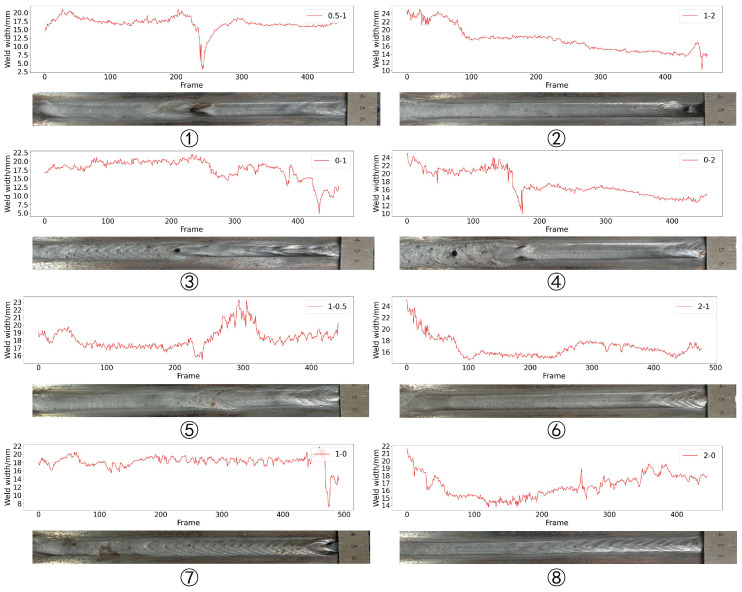
The results of weld pool width extraction.

**Figure 13:**
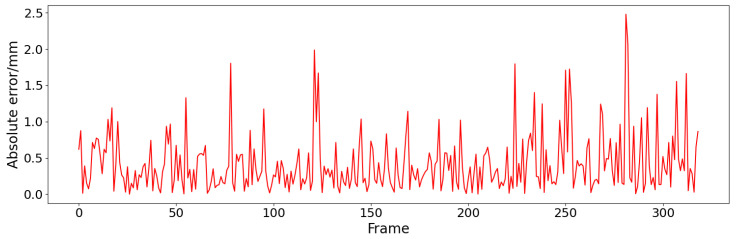
The absolute error of weld pool width extraction.

The width of the weld pool in the planar feature extraction result is compared with the width of the welding formation. The comparison result is shown in Figure 12. As can be seen, the trend in the extracted weld pool width is similar to the trend in the width of the welding formation. In order to evaluate the accuracy of feature extraction quantitatively, the label in the dataset is used to calculate the weld pool width as the reference. The absolute error between the reference and the weld pool width extraction is shown in Figure 13. The average absolute error is 0.41 mm and the maximum error is 2.47 mm.

#### 5.1.2. Three-Dimensional Weld Pool Reconstruction

**Figure 14:**
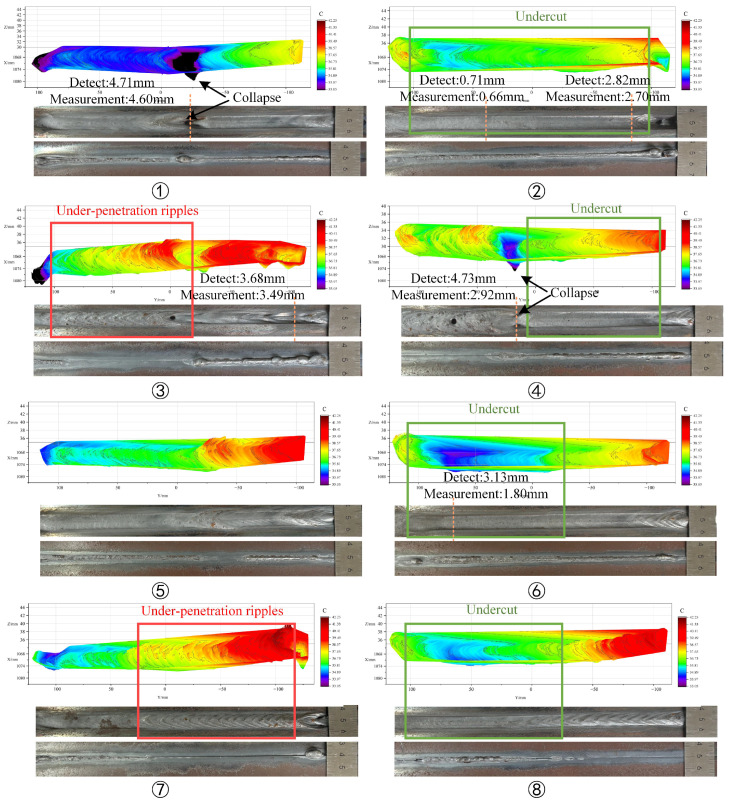
Comparison of three-dimensional morphology of weld pool and weld formation after welding in variable-gap K-TIG welding.

The three-dimensional contour of the weld pool is stitched using the robot coordinate information to obtain the three-dimensional morphology of the variable-gap K-TIG welding weld pool, as shown in Figure 14. The collapse, undercut, and under-penetration ripples of the weld formation can be clearly observed in the figure, but the porosity in group 3 and 4 cannot be accurately identified. Porosity is usually generated during the solidification process of the weld pool, resulting in failure of porosity observation for the stereo vision system. To evaluate the performance of the proposed 3D reconstruction of the weld pool contour algorithm, a vernier caliper is used to measure representative undercut and collapse positions to evaluate the accuracy of the three-dimensional reconstruction of the weld pool contour. As shown in Figure 14, it can be seen that the depth of the weld pool reconstructed by stereo vision is larger than the depth of the weld formation, with an average error of 0.60 mm. Moreover, the errors of group 4 and 6 are larger than the others. By comparing the collapse positions in group 1 and 3, it can be found that there is no serious overlap on the back formation of the undercut position in group 4 and 6. The molten metal is backfilled after the arc leaves this position, resulting in a large difference between the depth of the weld pool and the depth of the weld formation.

### 5.2. Extraction of Gap Width

**Figure 15:**
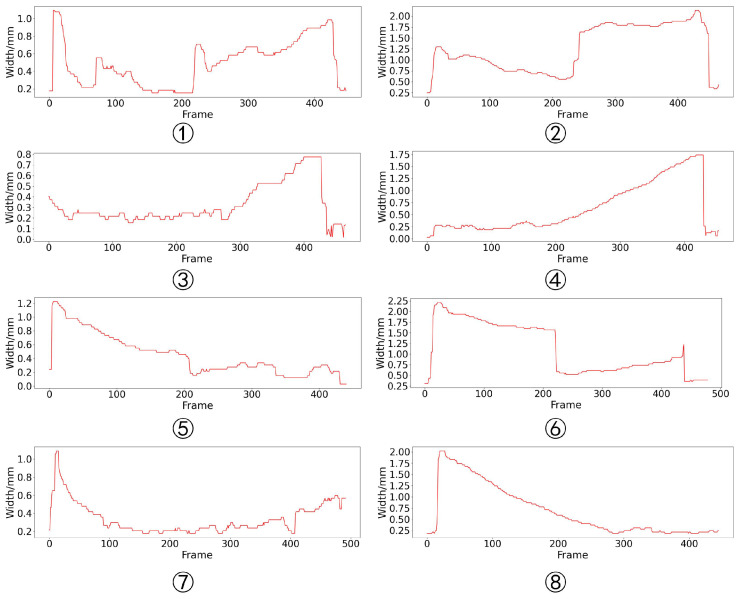
The results of gap width extraction.

**Figure 16:**
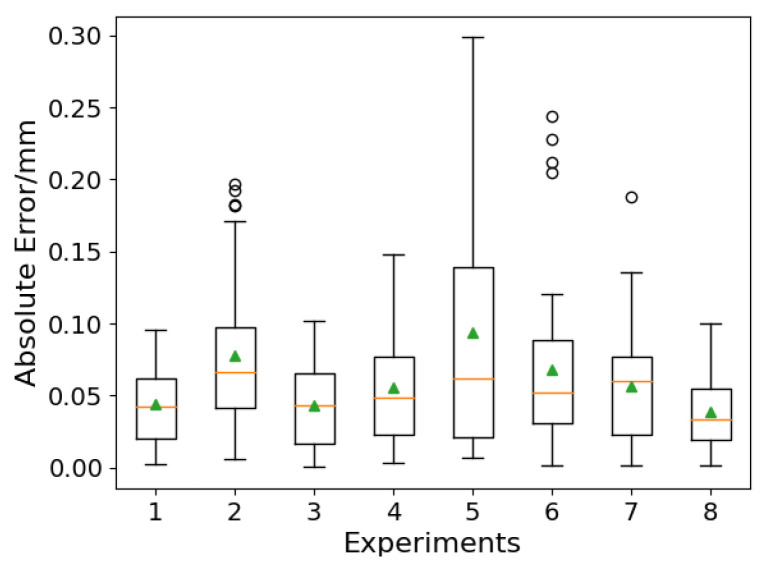
The box plot of seam width extraction.

The extraction result of gap width is shown in Figure 15. As can be seen, the gap width of most groups has a “bulge” phenomenon at the gap mutation position. This is because during the grinding process of the workpiece, the grinder tends to stay at the gap mutation position for a long time, causing the gap to expand. Although the gaps on two sides of the workpiece are less affected by grinding, the gap width of some groups still increases under the influence of welding thermal deformation. In order to evaluate the accuracy of the gap width extraction, it is necessary to determine the reference, but due to the influence of cutting and grinding accuracy and welding thermal deformation, the gap width is different from the gap width designed before welding. Therefore, the manual annotation method is used to obtain the reference gap width. One welding seam image is taken at intervals of 10 frames for annotation, and the absolute error between the annotation and the detection result is calculated. The evaluation results of each experiment are shown in Table 4. The average absolute error is 0.06 mm and the maximum error is 0.30 mm (group 5). The error box plot is shown in Figure 16. The triangles in the figure represents the average error and orange line represents the median error. As can be seen, the whiskers of groups 2, 4, and 5 are relatively long, indicating that the error distribution in these groups is wide and the stability of the algorithm is weak.

### 5.3. Penetration Recognition

In order to explore how the LSTM network recognizes the penetration state and understand the correlation between the geometric features of the K-TIG weld pool and the penetration state, an input feature ablation experiment was conducted. Different feature sets were selected to retrain the LSTM network and a test set that had never been used in the network training was used to evaluate the performance of different features. The results are shown in Table 5.

**Figure 17:**
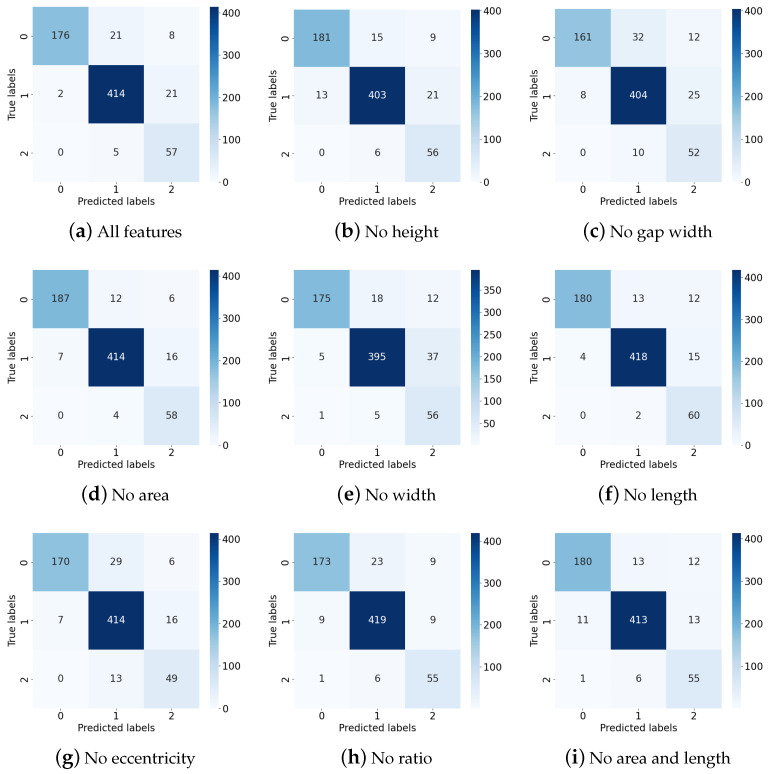
The confusion matrix for LSTM models based on different feature subsets.

As shown in Table 5, for the penetration state recognition model, the feature with the highest discrimination is the gap width, followed by the width, eccentricity, and height of the weld pool. In addition, the lack of area or length features increases the performance of the model. Therefore, a set of comparative experiments in which both area and length features are removed is added, and the accuracy of the trained model is 0.920. In order to further explore this phenomenon, the confusion matrix of each group is analyzed, as shown in Figure 17.

Comparing Figure 17a,b, it can be seen that with the absence of the height feature, the model will misclassify data that should be good penetration as under-penetration, which is consistent with the analysis in Section 3.1.2. In contrast, the width feature is of great importance for the model to distinguish good penetration and over-penetration. With the absence of width features, the probability of good-penetration data being misidentified as over-penetration increases, as shown in Figure 17e. Comparing Figure 17a,c, it is found that the gap width has an important influence on the recognition of all penetration states. Comparing Figure 17a,d,f,i, when the area or length feature is absent, the accuracy for under-penetration is improved. The lack of area feature is more conducive to the recognition of under-penetration, while the lack of length feature is more conducive to the recognition of good penetration. However, if both area and length features are absent, the recognition ability for the three penetration states will decrease. Comparing Figure 17a,g, the eccentricity feature has a promoting effect on the recognition of under-penetration and over-penetration states. As shown in Figure 17h, the keyhole-to-weld-pool-area ratio feature can improve the recognition ability for under-penetration.

**Figure 18:**
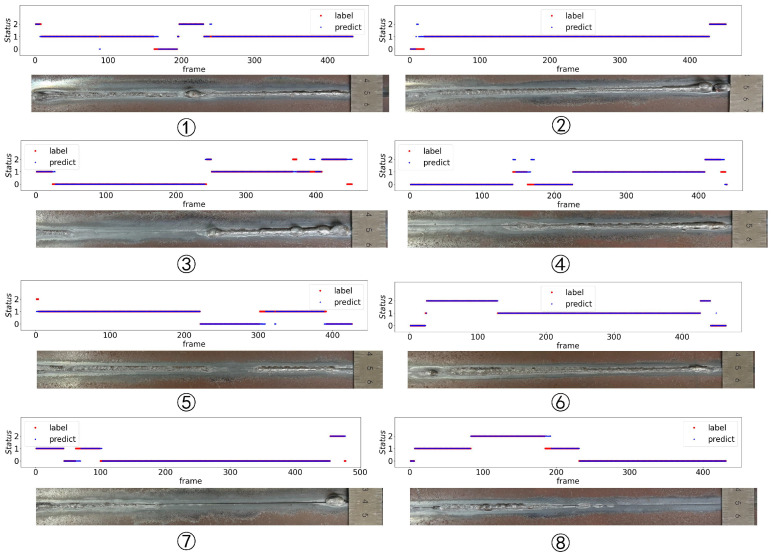
LSTM penetration state recognition results: 0—under-penetration; 1—good penetration; and 2—over-penetration.

According to the ablation experiment, the area feature should be removed. The accuracy of the penetration state recognition model in eight experiments is 0.967, as shown in Table 6. The results are shown in Figure 18.

According to Table 6, the accuracy of group 6 is the highest, while the accuracy of group 3 is the lowest. Combined with Figure 18, it can be found that misidentification is mainly concentrated in the position of the change of penetration state. The penetration state in group 3 changed most frequently, indicating that the “memory” of the LSTM network is a double-edged sword. It has the advantage of being able to handle the thermal inertia of K-TIG welding, but it finds it difficult to handle the frequent changes of the K-TIG penetration state because it relies on data before the current moment.

## 6. Conclusions

This paper aims to address the challenge of recognizing the penetration state in K-TIG welding caused by variations in the assembly gap of medium/thick plates. A penetration state recognition model based on 3D monitoring of the weld pool is proposed, wherein the 3D geometric features of the weld pool are extracted using a semantic segmentation network. The main contributions are as follows:

(1) An algorithm for extracting the planar features of the weld pool, as well as a stereo vision-based contour reconstruction algorithm for the weld pool using a semantic segmentation network, are proposed. The reconstructed 3D morphology of the weld pool clearly reveals phenomena such as collapse, undercut, and incomplete ripples in the formation. Additionally, the degree of collapse and undercut can be quantitatively assessed, achieving an average absolute error of 0.60 mm.

(2) A watershed-based gap width extraction algorithm is proposed. Experiments demonstrate that the average absolute error of the proposed algorithm is 0.06 mm and the maximum absolute error is 0.30 mm.

(3) The correlation between the extracted features and the penetration state is analyzed, revealing that gap width is the most significant feature influencing the accuracy of LSTM-based penetration state recognition, while the area feature acts as an interference variable. After excluding the area feature, the recognition accuracy of the model is 96.7%.

## Figures and Tables

**Table 1:** HDR camera parameters.

No.	Sensor Size	Resolution	FPS	Dynamic Range
LITS-FS3H	4.8 μm	1920 × 1080	30	120 dB

**Table 2:** Experimental parameters of 10 mm Q345R steel.

No.	Gap Width/mm	Gap Type	No.	Gap Width/mm	Gap Type
1	0.5-1	mutant	2	1-2	mutant
3	0-1	gradual	4	0-2	gradual
5	1-0.5	mutant	6	2-1	mutant
7	1-0	gradual	8	2-0	gradual

**Table 3:** The performance of BiSeNetV2.

Features	Training mIoU	Validation mIoU
Planar feature	0.963	0.931
Height feature	0.957	0.937

**Table 4:** The absolute error of gap width extraction experiments (unit: mm).

No.	Average Error	Maximum Error	No.	Average Error	Maximum Error
1	0.04	0.10	2	0.07	0.20
3	0.04	0.10	4	0.06	0.15
5	0.09	0.30	6	0.07	0.24
7	0.06	0.19	8	0.04	0.10

**Table 5:** Feature ablation experiments.

*H*	$W_{g}$	$A_{w}$	$W_{w}$	$L_{w}$	$E_{w}$	$A_{k}$	$W_{k}$	$L_{k}$	$E_{k}$	$K_{k/w}$	Test Accuracy
√	√	√	√	√	√	√	√	√	√	√	0.919
×	√	√	√	√	√	√	√	√	√	√	0.909
√	×	√	√	√	√	√	√	√	√	√	0.876
√	√	×	√	√	√	×	√	√	√	√	0.936
√	√	√	×	√	√	√	×	√	√	√	0.889
√	√	√	√	×	√	√	√	×	√	√	0.934
√	√	√	√	√	×	√	√	√	×	√	0.899
√	√	√	√	√	√	√	√	√	√	×	0.919

**Table 6:** The accuracy of penetration recognition.

No.	Accuracy	No.	Accuracy	No.	Accuracy	No.	Accuracy
1	0.970	2	0.973	3	0.935	4	0.952
5	0.962	6	0.993	7	0.972	8	0.976

## Data Availability

The dataset is available in https://drive.google.com/drive/folders/1fO1Lb6jumuDqYvAJFCVNE0Zmitw4_gOy?usp=drive_link (accessed on 25 November 2024).
